# Clinical pathologies of breast cancer in the elderly and youths and their prognosis

**DOI:** 10.12669/pjms.303.4929

**Published:** 2014

**Authors:** Hong Ji, Ning Ai, Qinghuai Li, Kaili Zhang, Wang Di

**Affiliations:** 1 Hong Ji, Department of General Surgery, The Second Hospital of Hebei Medical University, Shijiazhuang 050000, P. R. China.; 2 Ning Ai, Department of Radiology, The Fourth Hospital of Hebei Medical University, Shijiazhuang 050000, P. R. China.; 3 Qinghuai Li, Department of General Surgery, The Second Hospital of Hebei Medical University, Shijiazhuang 050000, P. R. China.; 4 Kaili Zhang, Department of General Surgery, The Second Hospital of Hebei Medical University, Shijiazhuang 050000, P. R. China.; 5 Wang Di, Department of General Surgery, The Second Hospital of Hebei Medical University, Shijiazhuang 050000, P. R. China.

**Keywords:** Age, Breast cancer, Immunohistochemistry, Pathological characteristics, Prognosis

## Abstract

***Objective:*** To explore the correlation between the clinical pathologies of breast cancer in the elderly and youths as well as their prognosis.

***Methods: ***Two hundred and eighty breast cancer patients were divided into a youth group (<60, n=120) and an elderly group (≥60, n=160) according to the age. Their routine clinical pathological indices and immune indices were observed and determined, and the prognosis was observed after effective treatment.

***Results: ***The positive expression rates of p63, CK5/6, CK14 and CK17 in the elderly group were significantly higher than those of the youth group (P<0.05). The tumor-free survival rate of the youth group (95.8%) was significantly higher than that of the elderly group (84.4%) (P<0.05). Multivariate Logistic regression analysis showed that the positive expressions of p63 and estrogen receptor, age, and postoperative chemotherapy were the independent risk factors of tumor-free survival rate (P<0.05).

***Conclusion: ***The immunohistochemical typing characteristics of the elderly and youths were different, and the prognosis of young patients was better, being correlated with the typing.

## INTRODUCTION

Breast cancer, which is malignant for women worldwide, has led to considerable morbidity and mortality rates even in youths.^1^^,^^2^ In China, the patients are mostly 40-49 years old. Since early diagnosis gives rise to satisfactory prognosis, it is crucial to diagnose and treat them as soon as possible to elevate the survival rate.^3^^-^^5^ In contrast, interim and advanced breast cancer patients usually require long-term treatment, thus tremendously burdening them and their families physically and physiologically, accompanied by high mortality rates.^6^ Recently, the expressions of estrogen receptor (ER) and progesterone receptor (PR) have been applied to determine the prognosis and treatment outcomes of breast cancer patients.^7^^-^^9^ Given that breast cancer is highly heterogeneous, the evaluation on early prognosis is subject to being affected by tumor metastasis, recurrence and surgical tolerance, etc.^10^

Generally, the patients older than 60 years old were prone to malignant tumors despite slow disease progression, whereas those younger than 60 commonly suffer from rapid progression. However, prognosis also evidently depends on the self-repair ability and immunity of different patients.^11^ Therefore, we herein explored the correlation between the clinical pathologies of breast cancer in the elderly and youths as well as the prognosis by determining the expressions of immune markers, clinical manifestations and prognosis.

## METHODS

***Subjects: ***All the experiments have been approved by the ethic committees of the Second Hospital of Hebei Medical University and the Fourth Hospital of Hebei Medical University, and written consent was obtained from all patients. Two hundred and eighty breast cancer patients enrolled in our hospitals from February 2008 to February 2013 were selected. ***Inclusion criteria***: Primary breast cancer; pathologically confirmed invasive non-specific cancer; unilateral disease; recurrence and metastasis confirmed by CT, B-mode ultrasound imaging and MRI; complete clinical and follow-up data; completion of radical or modified radical mastectomy; with signed written consent. ***Exclusion criteria:*** Completion of breast lump puncture one week before admission; completion of neoadjuvant chemotherapy; susceptible observation to inhomogeneous fat suppression; complicated with critical physical and mental diseases; older than 80 or younger than 18 years old. They were divided into a youth group (<60, n=120) and an elderly group (≥60, n=160) according to the age. Their tumor diameters, disease courses, tumor positions, lymphatic metastasis, TNM stages and family history did not differ significantly (P>0.05) (Table-I).

**Fig.1:**
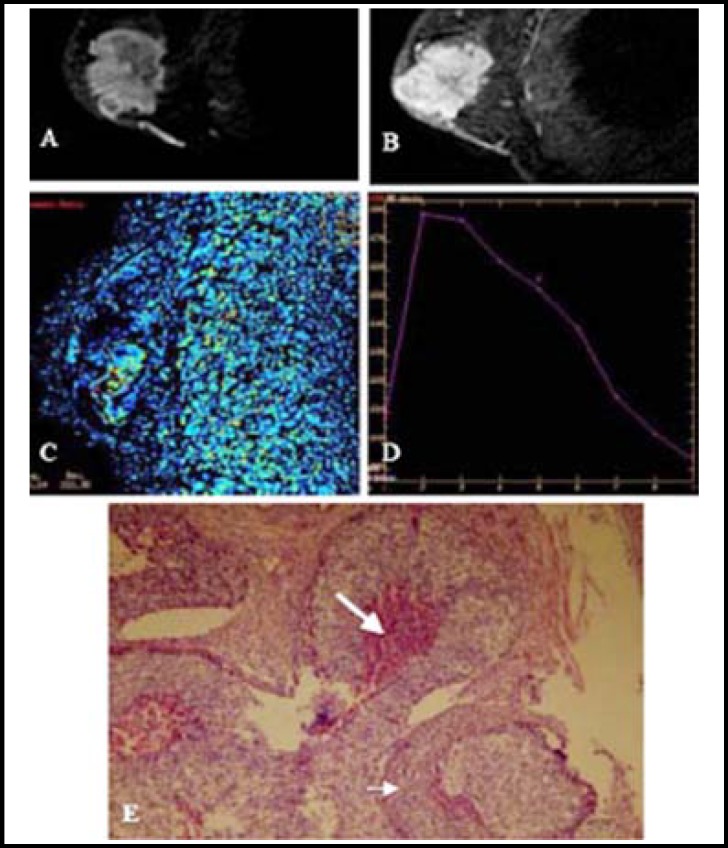
A 45-year-old female with invasive ductal carcinoma and lymphatic metastasis. **A): **Dynamic contrast-enhanced image; **B):** Subtraction image. A and B disclosed that the tumor focus was large. **C):** Enhancement rate >230%; **D):** Outflow enhancing mode and dynamic contrast-enhanced curve; **E):** Optical microscopic observation showed comedonecrosis (long arrow) and invasion of a large number of lymphocytes (short arrow) (HE×200).

**Fig.2:**
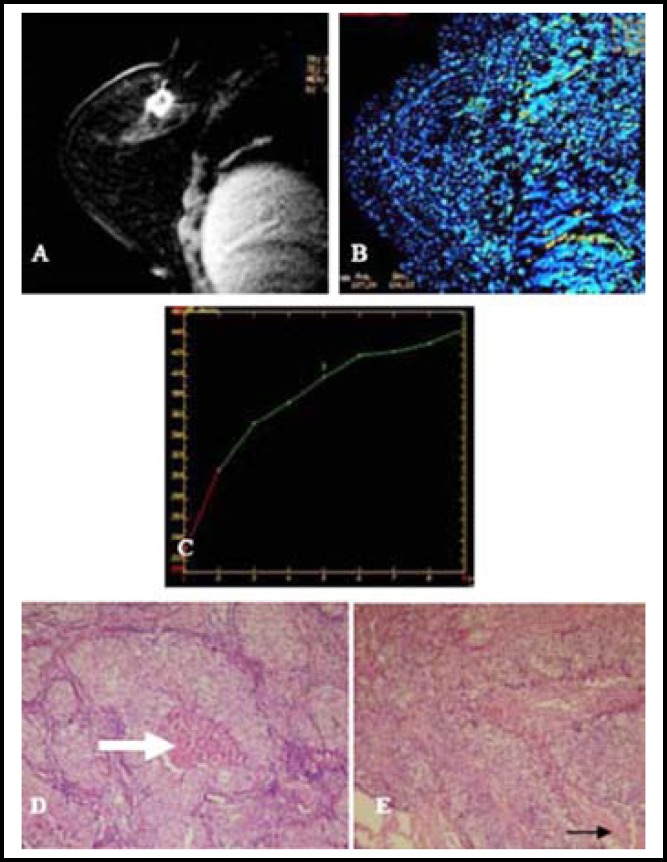
A 65-year-old female with invasive ductal carcinoma and without lymphatic metastasis. **A):** Dynamic contrast-enhanced image disclosed a ring-like tumor focus; **B):** Enhancement rate, 230%; **C):** Ascending enhancing mode and dynamic contrast-enhanced curve; **D and E):** Optical microscopic observation showed comedonecrosis (white arrow) and fibrosis (black arrow) (HE×100).

**Table-I:** Clinical data of the two groups

*Clinical data*	*Youth (n=120)*	*Elderly (n=160)*	*χ* ^2^ * or t*	*P*
Tumor size (cm)	4.56±0.32	4.62±0.44	0.324	>0.05
Disease course (month)	7.12±0.15	7.26±0.33	0.488	>0.05
Tumor position (left/right)	68/52	90/60	0.511	>0.05
Lymphatic metastasis (yes/no)	36/84	60/100	0.412	>0.05
TNM stage (I/II/III)	72/38/10	100/45/15	0.321	>0.05
Family history (yes/no)	24/96	32/128	0.000	>0.05

**Table-II:** Positive expression rates of immune markers in breast cancer patients (n).

*Immunohistochemical index*	*Youth (n=120)*	*Elderly (n=160)*	*χ* ^2^	*P*
Positive ER	56 (46.7%)	72 (45.0%)	0.225	>0.05
Positive PR	60 (50.0%)	79 (49.3%)	0.195	>0.05
Positive p53	36 (30.0%)	50 (31.3%)	0.188	>0.05
Positive p63	36 (30.0%)	102 (63.8%)	10.854	<0.05
Positive CK5/6	50 (41.7%)	100 (62.5%)	7.556	<0.05
Positive CK14	42 (35.0%)	121 (75.6%)	11.525	<0.05
Positive CK17	68 (56.7%)	144 (90.0%)	8.954	<0.05
Positive EGFR	61 (50.8%)	85 (53.1%)	0.369	>0.05

**Table-III:** Tumor-free survival rates of the two groups after treatment (n).

*Group*	*Case No.*	*Tumor-free survival for 5 years*	*5-year tumor-free survival rate*
Youth	120	115	95.8%
Elderly	160	135	84.4%
χ^2^			10.569
P			<0.05

**Table-IV:** Multivariate Logistic regression analysis of prognosis

*Risk factor*	*β*	*P*	*OR*	*95%CI*
Age	0.421	0.005	0.624	0.321-1.447
Positive p63	1.195	0.004	0.304	0.135-0.685
Positive ER	2.514	0.086	0.078	0.004-1.439
Postoperative chemotherapy	0.145	0.009	0.262	0.096-0.713

***Immunohistochemical analysis: ***Tumor samples were fixed in 10% neutral formalin and embedded in paraffin. The eligible sections were stained after 4μm continuous removal. PBS primary antibody, which was purchased from Beijing Zhongshan Jinqiao Biotechnology Co., Ltd., was utilized as the negative control. Known invasive breast cancer sections were used as the positive control. Mouse monoclonal antibodies (ER, PR, p53, p63, CK5/6, CK14, CK17, and VEFR) and immunohistochemical kits were bought from Fuzhou Maxim Biotech Inc. DAB chromogenic reagent was obtained from Sangon Biotech (Shanghai) Co., Ltd. All sections were observed under an optical microscope by a professional physiological doctor. ER, PR, p53 and p63 were positively expressed in cell nucleus, while CK5/6, CK14 and CK17 were positively expressed in cytoplasm, and EGFR was positively expressed in cell membrane.

***Treatment methods: ***All patients received radical or modified radical mastectomy, of which 88 in the youth group and 110 in the elderly group received postoperative chemotherapy, and 50 in the youth group and 65 in the elderly group received radiotherapy at chest wall, regional lymph nodes and internal mammary area. They were follow-up until November, 2013 with the contents regarding disease course, tumor diameter, lymphatic metastasis, TNM stage, chemotherapy, radiotherapy, immunohistochemical expression and survival.

***Statistical analysis: ***All data were analyzed by SAS13.0. The pathological characteristics and routine data were compared by χ^2^ or t test. Multivariate analysis was performed by using Logistic regression model corrected confounding factors. P<0.05 was considered statistically significant.

## RESULTS

***Comparison between immunohistochemical analysis results: ***The positive expression rates of ER, PR, EGFR and p53 in the two groups did not differ significantly (P>0.05), whereas the rates of p63, CK5/6, CK14 and CK17 in the elderly group were significantly higher than those of the youth group (P<0.05) (Table-II).

***Case analysis: ***Two cases from the two groups were analyzed (Fig. 1 and 2).

***Prognosis analysis: ***Until 30th November 2013, a total of 250 patients had the tumor-free survival times ≧5 years, of which there were 115 from the youth group and 135 from the elderly group. The tumor-free survival rates of the youth group and the elderly group were 95.8% and 84.4% respectively, with a significant difference (P<0.05) (Table-III).

***Risk factors affecting prognosis: ***By using multivariate Logistic regression analysis, the positive expressions of p63 and ER, age, and postoperative chemotherapy were screened as the independent risk factors of tumor-free survival rate (P<0.05) (Table-IV).

## DISCUSSION

Breast cancer is the most devastating malignant tumor that threatens women worldwide.^12^ Many Chinese women, especially those in metropolis, are prone to breast cancer even at young age owing to active or passive uptake of endogenous and exogenous estrogen with the rapid economic and social development, habitual changes, environmental contamination and working pressure.^13^ Currently, approximately 40 out of 100 thousand women in China are suffering from breast cancer. Generally, the patients older than 60 years old were prone to malignant tumors despite of slow disease progression, whereas the younger ones were commonly subjected to rapid progression.^14^

It is well known that breast cancer is highly heterogeneous, thus rendering diagnosis and treatment tricky with complex clinical manifestations, pathological characteristics and prognosis.^15^ The tumor diameters, disease courses, tumor positions, lymphatic metastasis, TNM stages and family history of the two groups in this study did not differ significantly (P>0.05). To improve the prognosis specifically, indices such as ER and PR have been applied to determine the onset, progression and selection of treatment protocols. It has previously been reported that the breast cancer of patients younger than 35 years old is more invasive, leading to poor prognosis.^16^ In this study, the positive expression rates of ER, PR, EGFR and p53 in the two groups did not differ significantly (P>0.05), whereas the rates of p63, CK5/6, CK14 and CK17 in the elderly group were significantly higher than those of the youth group (P<0.05). The expressions of CK5/6 and CK17 are independent prognostic factors of breast cancer, without being affected by tumor size or axillary lymph node metastasis.

 The tumor-free survival rates of the youth group and the elderly group were 95.8% and 84.4% respectively, with a significant difference (P<0.05). As a member of p53 gene family, p63 is highly expressed in the basal cell layer of normal tissue epithelia, thus evidently influencing the proliferation of basal stem cells and epithelial tissues. The expression of p63 is associated with tumor size, histological grade, lymphatic metastasis and negative ER expression. Of the patients receiving surgeries, higher expression of p63 gave rise to better prognosis. Meanwhile, some breast cancer patients who had high contents of endogenous estrogen in blood and low ER expression were more subject to death due to distant metastases and recurrence.^17^

In summary, the immunohistochemical typing characteristics of the elderly and youths were different, and the prognosis of young patients was better.

## Authors Contributions:

**HJ and NA** **:** Designed the protocol and prepared the final manuscript.**QL, KZ and WD: **Clinical data collection and experiments.
